# Monoclonal Gammopathy of Neurological Significance in a Patient With Chronic Lymphocytic Inflammation With Pontine Perivascular Enhancement Responsive to Steroids (CLIPPERS Syndrome): A Case Report of a Rare Entity

**DOI:** 10.7759/cureus.67386

**Published:** 2024-08-21

**Authors:** Andres E Prieto-Torres, Angela S Esparza-Albornoz, Nohora A Ovalle-Roa, Ivan Pisciotti-Chajin, Humberto Martinez-Cordero

**Affiliations:** 1 Internal Medicine, Hospital Militar Central, Bogotá, COL; 2 Hematology and Oncology, Hospital Militar Central, Bogotá, COL; 3 Hematology, Hospital Militar Central, Bogotá, COL; 4 Oncology, Hospital Militar Central, Bogotá, COL; 5 Hematology, Instituto Nacional de Cancerología, Bogotá, COL

**Keywords:** clippers syndrome, dexamethasone, lenalidomide, paraproteinemia, monoclonal gammopathies of neurological significance (mgns)

## Abstract

Paraproteinemias or monoclonal gammopathies constitute a broad spectrum of heterogeneous clonal disorders of plasma cells characterized by the secretion of monoclonal proteins of heavy or light chains and the development of symptoms associated with them through mechanisms independent of tumor burden. Specifically, peripheral neuropathies represent an increasingly recognized manifestation of these paraproteinemias.

We report a case of a 71-year-old female who presented to the emergency department with clinical symptoms of perioral paresthesias associated with an ataxic gait that progressively compromised her functionality, eventually completely limiting her ability to walk. Initially diagnosed with chronic lymphocytic inflammation with pontine perivascular enhancement responsive to steroids (CLIPPERS syndrome), management with corticosteroids was initiated, leading to partial improvement. After comprehensive etiological studies ruled out common causes of peripheral neuropathy (PN), a monoclonal peak of immunoglobulin M (IgM) was detected. With the initiation of appropriate treatment, the patient progressively regained her ability to walk. Unfortunately, due to prolonged corticosteroid use, she developed osteoporosis and multiple fragility fractures, which again limited her mobility. CLIPPERS syndrome coexisting with monoclonal gammopathy is extremely rare, highlighting the importance of this report.

## Introduction

Monoclonal gammopathies, or paraproteinemias, encompass a heterogeneous spectrum of clonal plasma cell disorders ranging from monoclonal gammopathy of undetermined significance (MGUS) and smoldering multiple myeloma (SMM) to symptomatic multiple myeloma, POEMS syndrome (polyneuropathy, organomegaly, endocrinopathy, monoclonal plasma cell disorder, and skin changes), solitary plasmacytoma (SP), and primary systemic amyloidosis. These disorders are characterized by the secretion of monoclonal proteins of heavy or light chains and the development of symptoms related to clones through mechanisms other than tumor burden [1]. Given the specific disease features due to the abnormal clonotypic immunoglobulin, which can be detected as a serum biomarker at the preclinical level, the classification of these neoplasms requires the integration of clinical and laboratory features for staging and therapy decisions [2].

Specifically, MGUS are benign conditions with malignant potential and can be classified into two distinct groups: non-immunoglobulin M (IgM) MGUS (IgG, IgA, and MGUS - kappa and lambda) and IgM MGUS. In the natural course of the disease, most patients with non-IgM MGUS tend to develop multiple myeloma (MM) or systemic light chain (AL) amyloidosis, while most patients with IgM MGUS progress to Waldenström macroglobulinemia or other lymphoproliferative disorders [1]. The diagnosis of IgM MGUS is established in cases of IgM paraprotein with <10% bone marrow clonal plasma cells and a lack of lymphoplasmacytic B-cell aggregates sufficient for the diagnosis of lymphoplasmacytic lymphoma [2].

The association between MGUS and peripheral neuropathy (PN) has been established in population studies, where an increased relative risk was evident, but with the caveat that the relationship between these may be non-causal [3]. PNs are a well-known manifestation of paraproteinemias and become more prevalent as the population ages (3-4% above the age of 50 years; over 5% in patients over 70 years) and represent 3-5% of all PNs. In 50% of cases, there is a causal association, while the rest is likely coincidental. Peripheral nerve involvement is not considered a terminal organ involvement of plasma cell disorders, and there is no consensus on whether to initiate therapy [4].

The most commonly associated monoclonal paraprotein with peripheral neuropathy is IgM, followed by IgG and IgA. Clinical and electrophysiological characteristics are more heterogeneous in IgG/IgA than in IgM and may be indistinguishable from chronic idiopathic demyelinating polyneuropathies (CIDP). There are no specific antibodies associated with IgG/IgA demyelinating PN, and testing for anti-ganglioside antibodies or antibodies to myelin-associated glycoproteins (MAG) is not required [5]. Axonal PN often occurs in MGUS IgA/IgG patients, but there is no clear causal relationship except in patients with AL amyloidosis and POEMS syndrome. Biopsy in these cases, unlike its utility in monoclonal gammopathies with renal significance (MGRS), may be associated with permanent sensory or motor deficits and distal pain, making it not useful (pathology shows demyelination and widening of myelin lamellae with deposits usually of monoclonal IgM and myelin debris in Schwann cells and macrophages) [6].

On the other hand, CLIPPERS syndrome is an inflammatory disease that occurs around the pons, midbrain, and cerebral vessels, characterized primarily by lymphocytic infiltration and is generally effectively treated with steroids. It usually manifests between the ages of 30 and 60 years, with a male preponderance [7]. There are no reports in the literature on the coexistence of MGUS with CLIPPERS syndrome, making this case report extremely rare and interesting.

## Case presentation

**Figure 1:**
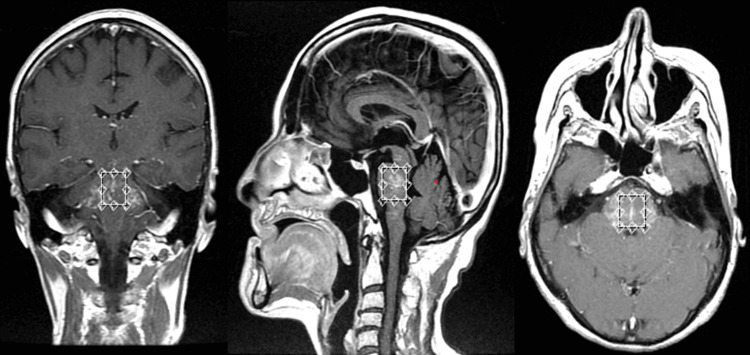
MRI of the patient MRI showing hyperintense images at the level of the midbrain, pons, and both cerebellar peduncles and medulla, revealing isointense behavior in T1 with avid post-contrast enhancement MRI: magnetic resonance imaging

A 71-year-old woman, with a relevant history of asthma, presented with clinical symptoms of perioral paresthesias, right facial paralysis, diplopia, and gait instability. Her medical history from an external institution included an MRI scan showing T2/FLAIR hyperintense images at the level of the midbrain, pons, and both cerebellar peduncles and medulla, revealing isointense behavior in T1 with avid post-contrast enhancement (Figure 1). A right peripheral paralysis was confirmed, associated with diplopia with paralysis of the left sixth cranial nerve, and an ataxic gait, all consistent with findings described in the initial imaging; she was treated with acyclovir 800 mg five times daily plus prednisolone 50 mg for seven days. The lab findings are shown in Table 1.

**Table 1:** Lab findings MCV: mean corpuscular volume; MCH: mean corpuscular hemoglobin; EDW: erythrocyte distribution width; HIV: human immunodeficiency virus; HCV: hepatitis C virus; IgG/IgM/IgA: immunoglobulin G/M/A; CSF: cerebrospinal fluid

Parameter	Result	Normal range
Blood count		
Leukocytes	7.76 x 10^3^/mm^3^	(4.5 – 11.3)
Neutrophils	3.68 x 10^3^/uL	(2.25 – 8.48)
Lymphocytes	3.11 x 10^3^/uL	(0.9 – 4.52)
Eosinophils	0.07 x 10^3^/uL	(0 – 0.45)
Basophils	0.02 x 10^3^/uL	(0 – 0.11)
Monocytes	0.81 x 10^3^/uL	(0 – 1.24)
Hemoglobin	10.7 g/dl	(12.1 – 15)
Hematocrit	34%	(35 – 44)
MCV	87.7 fl	(80 – 100)
MCH	27.8 pg	(23 – 34)
EDW	13.2%	(11.5 – 14.5)
Platelets	371 x 10^3^/uL	(150 – 450)
Others		
Calcium	9.2 mg/dl	(8.6 – 10)
Uric acid	2.2 mg/dl	(2.4 – 5.7)
Albumin	4.3 gr/dl	(3.9 – 4.9)
Total proteins	6.36 gr/dl	(6.6 – 8.7)
Ureic nitrogen	14.1 mg/dl	(8 – 23)
Creatinine	0.70 mg/dl	(0.51 – 0.95)
Prothrombin time (PT)	10.3 seconds	(10.7)
Thromboplastin time (PTT)	21.6 seconds	(28)
B12 vitamin	230.5 pg/ml	(197 – 771)
HIV antibodies	Non-reactive	-
HCV antibodies	Non-reactive	-
Beta 2 microglobulin	1.14 mg/L	(0.8 – 2.34)
Lupus anticoagulant	Negative	-
Anticardiolipin antibodies IgG/IgM	Negative	-
Extractable antinuclear antibodies (ENAs)	Negative	-
Antinuclear antibodies (ANAs)	1/80 positive	-
Serum complement (C3)	112 mg/dl	(81.1 – 157)
Serum complement (C4)	17.30 mg/dl	(12.9 – 39.2)
Rheumatoid factor	5.7 UI/ml	(0 – 12.5)
Antineutrophil cytoplasmic antibodies (ANCAs)	Negative	-
Serum copper cadmium and lead	Not detected	-
Neuromyelitis optic IgG antibodies	Negative	-
Anti-gangliosides antibodies in CSF	Negative	-
Cryoglobulins	Negative	-
Protein electrophoresis	Monoclonal peak in gamma zone	-
Serum immunofixation	IgM monoclonal appearance band, kappa light chain is characterized	-
Serum-free kappa light chain	71.37 mg/L	(3.3 – 19.4)
Serum-free lambda light chain	5.32 mg/L	(5.71 – 28.3)
Ratio kappa/lambda calculated	13.41	-
Serum immunoglobulin IgM	824 mg/dl	(35 – 242)
Serum immunoglobulin IgA	90 mg/dl	(84.5 – 499)
Serum immunoglobulin IgG	773 mg/dl	(610 – 1616)

**Figure 2:**
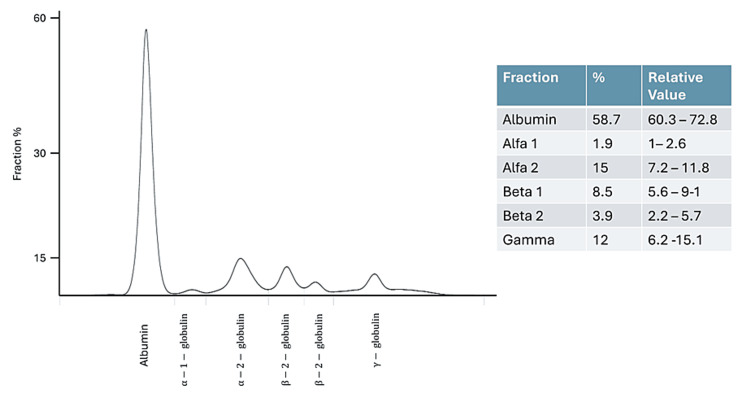
Protein electrophoresis The image shows abnormal morphology in the gamma zone suggestive of a monoclonal peak. Immunofixation revealed an IgM monoclonal peak. The serum kappa/lambda ratio revealed an abnormal production of kappa light chains

Considering a possible inflammatory etiology of findings on brain MRI, a lumbar puncture was performed, which showed no alterations. Serum anti-AQP4 antibodies, blood-heavy metals levels, and anti-ganglioside GQ1B antibodies in cerebrospinal fluid were all negative. To rule out metastasis, additional imaging was requested; chest tomography revealed four pulmonary nodules, and lobectomy revealed a non-malignant pathology. Given the normal volume anemia, protein electrophoresis was indicated, showing an altered morphology in the gamma zone suggestive of a monoclonal peak. Serum immunofixation revealed a monoclonal IgM heavy chain band and a kappa light chain. The serum kappa/lambda ratio was abnormally elevated, which could only be due to a plasma cell proliferative (or lymphoproliferative) disorder that secretes excess free light chains (FLC) and disturbs the normal balance between kappa and lambda secretion (Figure 2).

The rheumatology service considered the possibility that the condition corresponded to giant cell arteritis, performing a normal temporal artery Doppler and a negative autoimmune profile except for ANAs positive at 1/80 (not relevant due to their high prevalence in the general population within that range). Empirically, due to suspicion of an inflammatory etiology, treatment with oral steroids and methotrexate was initiated, which was gradually discontinued once the analyses were completed. After nearly complete tapering of the steroid dose and one year after symptom onset, there was clinical deterioration with progression of diplopia, dysarthria, and headache. Prednisolone at 1 mg/kg was initiated due to suspicion of CLIPPERS syndrome based on imaging reports and clinical presentation.

After eight months, the patient's systemic symptoms persisted, with progressive weight loss. Initially, a bone marrow biopsy showed 8% plasma cells without light chain restriction; a second biopsy revealed 15% plasma cells positive for CD38 and CD138 markers with aberrant loss of CD19 and restriction of kappa/lambda light chains. Approximately two years after symptom onset, the patient had a hematology consultation in a wheelchair with the latest bone marrow report, which led to a diagnosis of IgM kappa MM, with an indication for treatment initiation with RVD lite regimen (bortezomib, lenalidomide, and dexamethasone) in 35-day cycles. However, after a few cycles, neurological deterioration was observed, likely related to the use of bortezomib. A new bone marrow biopsy showed no monoclonal plasma cells, raising the possible diagnosis of monoclonal gammopathy of neurological significance, with a high risk for developing MM due to the kappa/lambda ratio as a prognostic factor for progression.

Medical treatment was adjusted with the FIRST protocol using oral lenalidomide 15 mg on days 1-21 and oral dexamethasone 20 mg on days 1, 8, 15, and 22. Six months later, the patient began walking again without any limitation, with a concomitant decrease in serum IgM levels and normalization of the kappa/lambda ratio. Electromyography with nerve conduction studies revealed axonal sensory-motor polyneuropathy in all four extremities, which neurology regarded as consistent with the diagnosis proposed by hematology. Unfortunately, chronic exposure to steroid use in the patient led to the development of osteoporosis and secondary fractures in some vertebral bodies and the right hip. Currently, the patient is undergoing supplementation with calcium, vitamin D, and teriparatide, along with physical rehabilitation following hip surgery.

## Discussion

Peripheral neuropathies associated with IgM typically manifest as sensory rather than motor symptoms, featuring symmetrical distribution, length dependence, and slow progression. The specific clinical syndrome in this case is distal acquired demyelinating symmetric neuropathy with monoclonal gammopathy (DADS-M), which must be distinguished from CIDP [8]. Its progression is insidious, with sensory ataxia being the most common sign, as seen in our patient; significant disability can develop in up to 50% of patients 10-15 years after the diagnosis. While about 50% of DADS-M patients have elevated anti-MAG levels, this does not correlate with the severity of PN. In DADS-M patients negative for anti-MAG (50-60%), evaluation for anti-ganglioside antibodies (35%) and paragloboside glucuronyl 3 sulfate antibodies (9%) (SGPG) is warranted [9].

We conducted comprehensive investigations to exclude common diseases that could cause neuropathies, including chronic alcohol consumption, diabetes, vitamin B12 deficiency, HIV, and autoimmune conditions. Initially, due to IgM monoclonality, one of the main differentials considered was Waldenström's macroglobulinemia [10]. However, the absence of associated features such as hemolytic anemia, thrombocytopenia, organomegaly, and lymphadenopathy weakened the chances of this diagnosis and practically ruled out Bing-Neel syndrome, a condition characterized by lymphoplasmacytic lymphoma cells accessing the central nervous system, causing neurological deficits [11].

Even less common etiologies such as cryoglobulinemias were appropriately ruled out, and a negative Congo red staining on fat pad biopsy excluded AL amyloidosis based on the mixed characteristics described in the electromyographic study. We also considered the possibility of neuropathies mediated by anti-MAG antibodies or anti-ganglioside antibodies. Consequently, our differential diagnosis was limited to pure anti-MAG neuropathy versus CANOMAD (chronic ataxic neuropathy with ophthalmoplegia, monoclonal gammopathy, cold agglutinins, and anti-disialosyl ganglioside antibodies), a rare condition exhibiting mixed demyelinating and axonal characteristics on electromyography [12]. Given the unavailability of specific antibodies, our diagnostic approach was constrained regarding the evaluation of certain neuropathic conditions mediated by these antibodies. Our strategy focused on properly ruling out other known causes of PN through available clinical tests and laboratory studies. Hence, medical management was initiated with the FIRST protocol, leading to an adequate response in the patient, who progressively began to walk again.

Interestingly, our patient received treatment from the beginning with corticosteroids, initially due to concerns about an autoimmune phenomenon such as vasculitis and subsequently CLIPPERS syndrome, based on highly suggestive characteristics on diagnostic imaging [7]. However, the lack of a complete response to steroids, intolerance, and progression with dose reduction, along with the complete response to the implementation of our management protocol, strongly suggest that we are dealing with MGNS. To date, there have been no reports of CLIPPERS syndrome associated with or related to monoclonal gammopathies; they have never been described within the hypotheses of its pathophysiology either, which makes it intriguing to explore the possible relationship between the two conditions.

## Conclusions

Monoclonal gammopathies with associated neuropathy are constantly evolving, requiring further studies to define the possible pathologies to aid the investigation into their etiological diagnosis. They should be differentiated, for example, from plasma cell disorders such as AL amyloidosis and POEMS syndrome, where the causal relationship between the M protein and the neurological clinical presentation is undeniable. Additionally, this case demonstrates that in the absence of specific antibodies for diagnosis, clinical presumption is sufficient to initiate treatment. Even though the case presents evidence of the coexistence of two conditions, it does not establish a causal relationship between them. Further research is required to establish the generalizability of our findings.
